# Cholera public health surveillance in the Republic of Cameroon-opportunities and challenges

**DOI:** 10.11604/pamj.2016.24.222.8045

**Published:** 2016-07-12

**Authors:** Moise Chi Ngwa, Song Liang, Leonard Mbam Mbam, Arabi Mouhaman, Andrew Teboh, Kaousseri Brekmo, Onana Mevoula, John Glenn Morris

**Affiliations:** 1 Emerging Pathogens Institute, University of Florida, Gainesville, Florida, USA; 2 Department of Environmental and Global Health, College of Public Health and Health Professions, University of Florida, Gainesville, Florida, USA; 3 World Health Organization country office for the Republic of Cameroon, Yaoundé, Cameroon; 4 Department of Environmental Sciences, Higher Institute of the Sahel, University of Maroua, Maroua, Cameroon; 5 Central Africa Field Epidemiology and Laboratory Training Program, Faculty of Medicine and Biomedical Sciences, University of Yaoundé I, Yaoundé, Cameroon; 6 Regional Public Health Delegation for the Far North Region, Maroua, Cameroon; 7 World Health Organization country office, Far North Region, Maroua, Cameroon; 8 Department of Medicine, College of Medicine, University of Florida, Gainesville, Florida, USA

**Keywords:** Cholera, Integrated Disease Surveillance Response strategy, surveillance, Cameroon

## Abstract

**Introduction:**

In Cameroon, cholera has periodically resurfaced since it was first reported in 1971. In 2003, Cameroon adapted the Integrated Disease Surveillance and Response (IDSR) strategy to strengthen surveillance in the country. This study was an in-depth description and assessment of the structure, core and support functions, and attributes of the current cholera surveillance system in Cameroon. It also discussed its strengths and challenges with hope that lessons learned could improve the system in Cameroon and in other countries in Africa implementing the IDSR strategy.

**Methods:**

Semi-structured key informant interviews, peer reviewed articles, and government record review were conducted in the Far North and Centre Regions of Cameroon. We used the matrix and conceptual framework from the World Health Organization (WHO) and Centers for Disease Control and Prevention, WHO Regional Office for Africa Technical Guidelines to frame the study. Site visits included the WHO country office, the ministry of public health (MoPH), two Regional Public Health Delegations (RPHDs), eight health districts (HDs) and health facilities (HFs) including two labs.

**Results:**

Cholera surveillance is passive but turns active during outbreaks and follows a hierarchical structure. Cholera data are collected at HFs and sent to HDs where data are compiled and sent to the RPHD in paper format. RPHDs de-identify, digitalize, and send the data to the MoPH via internet and from there to the WHO. The case definition was officially changed in 2010 but the outdated definition was still in use in 2013. Nationally, there are 3 laboratories that have the ability to confirm cholera cases; the lack of laboratory capacity at HFs hampers case and outbreak confirmation. The absence of structured data analysis at the RPHD, HD, and HF further compounds the situation, making the goal of IDSR of data analysis and rapid response at the HD very challenging. Feedback is strongest at the central level (MoPH) and non-existent at the levels below it, with only minimal training and supervision of staff. In 2012, mobile phone coverage expanded to all 183 HDs and to HFs in 2014 in the Far North and North Regions. The phones improved immediate reporting and outbreak control. Further, the creation of cholera command and control centers, and introduction of laptops at all RPHDs are major strengths in the surveillance system. Completeness and timeliness of reporting varied considerably among levels.

**Conclusion:**

Significant milestones in the hierarchical structure towards integration and achieving early detection and rapid response in cholera surveillance are in effective use; however, some challenges exist. The surveillance system lack labs at HFs and there is no data analysis at HD level. Thus, the goal of IDSR-strategy of early detection, data analysis, and rapid response at the HD level is a challenge. Both human and material resources are needed at the HD level to achieve this goal.

## Introduction

Toxigenic strains of the comma-shaped bacterium *Vibrio cholera* cause cholera, a disease characterized by severe watery diarrhea. This bacterium secretes cholera toxin that binds to host enterocytes leading to massive loss of water and electrolytes in profuse diarrhea [1]; if left untreated, patients with the most severe form of the disease can become dehydrated and die in a matter of hours. There are > 200 serogroups of *V. cholera*, and of these only the O1 and O139 have been linked with epidemic disease [2].

Seven *V. cholera* pandemics have been recognized since 1817 [1], with the seventh that originated from the Celebes in 1961 ongoing [3, 4]. In 1970, the latter pandemic reached the continent of Africa first in the West African countries of Guinea-Bissau [5], and Guinea Conakry, and then reached Central Africa, in Cameroon, in 1971 [6]. Without any cholera reported prior to 1970, the continent bore the brunt of the global cholera caseload, between 2000 and 2012 [7]. In this period, the following cases were reported to the World Health Organization (WHO): Africa 1,977,808; Asia 118,038; Americas 665,086; Oceania16, 286; and Europe 392 [7]. Further, the four countries around the Lake Chad Basin (Niger, Nigeria, Chad, and Cameroon) reported 62,762 cases in 2010; 65,401 in 2011; and 6,784 in 2012 to the WHO [7]. Of these, Cameroon reported 22,762 cases including 786 deaths in 2011 alone [8]. While it may be anticipated that actual case numbers are substantially higher than reported cases [9], an accurate assessment of the reliability of these data requires an understanding of the strengths and weaknesses of the underlying national cholera surveillance system [10].

Prior to the year 2000, data on other epidemic-prone diseases in Africa were not reported in a timely manner, with problems in data analysis and dissemination [9–12]. While vertical surveillance systems, which involve data collection linked to various disease intervention programs such as malaria, and HIV/AIDS, had been established, such systems were inflexible and duplicated resources. Consequently, the 48^th^ WHO-AFRO Committee for Africa in 1998 adopted a strategy called Integrated Disease Surveillance (IDS) [11]; which was latter referred to as the Integrated Disease Surveillance and Response (IDSR) strategy to emphasize the critical link between surveillance and response. This strategy was designed to strengthen early detection and efficacious response to infectious diseases in the African region. Thus, IDSR aimed to integrate all surveillance core functions and response core functions at all levels of health system with particular focus at the health district (HD) level. Further, IDSR aimed at streamlining and directing all surveillance support functions to the HD level [12, 13]. The goal of the WHO-AFRO-IDSR strategy is to integrate these core, and support functions at the central, regional, HD, health facility (HF), and community levels in an action-oriented surveillance system with response at the HD level [12].

The first edition of the IDSR Technical Guidelines (2001) suggested 19 communicable diseases and conditions for integration. However, owing to changes in disease landscape since 2001, the WHO and Centers for Disease Control and Prevention (CDC) Atlanta, in 2010 developed the second edition of the IDSR Technical Guidelines with 43 diseases and conditions/events for integration [14]. Member States effecting IDSR must consider changes in the second edition and the International Health Regulation (IHR) (2005), which called for the strengthening of national public health surveillance systems [15]. A survey in 2010 showed that 43 out of the 46 members were at various stages of IDSR implementation, and that 3,801 health districts out of 4,386 in 45 countries have implemented some form of IDSR strategy [16]. As a model for strengths and weaknesses in these systems, we will look at the IDSR strategy activities in The Republic of Cameroon (Cameroon) with a particular focus on cholera.

Cameroon first adopted the IDSR strategy in 2003 [17], thus ending the single disease program that relied specifically on the vertical public health surveillance systems. The first and second editions of the Technical Guidelines were implemented in 2005 [18], and 2011 [19], respectively. In adopting the second edition Technical Guidelines (2010), Cameroon added 4 more diseases to the 43 proposed by the generic version, resulting in 47 diseases under IDSR surveillance in the country (see supplemental Table S1) [20].

## Methods

### Study setting

**Figure 1:**
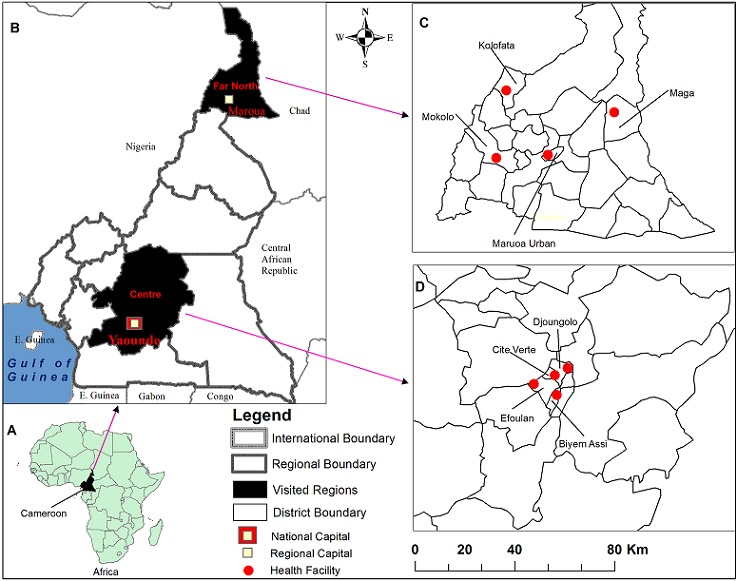
Study setting for the IDSR strategy study

Geographically, Cameroon is located on the Gulf of Guinea between latitude 2^nd^ and 13^th^ degrees north and longitude 9th and 16th degrees east (Figure 1A). Figure 1B shows the countries that border Cameroon with a coastline along the Atlantic Ocean. We conducted site visits in the Far North and Centre Regions (Figure 1B), which were heavily affected by the latest cholera outbreaks of 2009-2012. Demographic statistics show 3,669,600 (population density107.1 inhabitants/km2), and 3,730,800 (population density 54 people/km²) inhabitants in the Far North and Centre regions, in 2012, respectively [21]. The former is divided into 30 HDs, and of these four (one urban and three rural) were visited including four HFs (Figure 1C) and two laboratories. The Centre Region is host to the national capital Yaoundé (Figure 1B) and a number of key institutions including the Ministry of Public Health (MoPH), and WHO-country office. We visited four HDs and HFs in the Centre Region (Figure 1D).

### Study design

**Figure 2:**
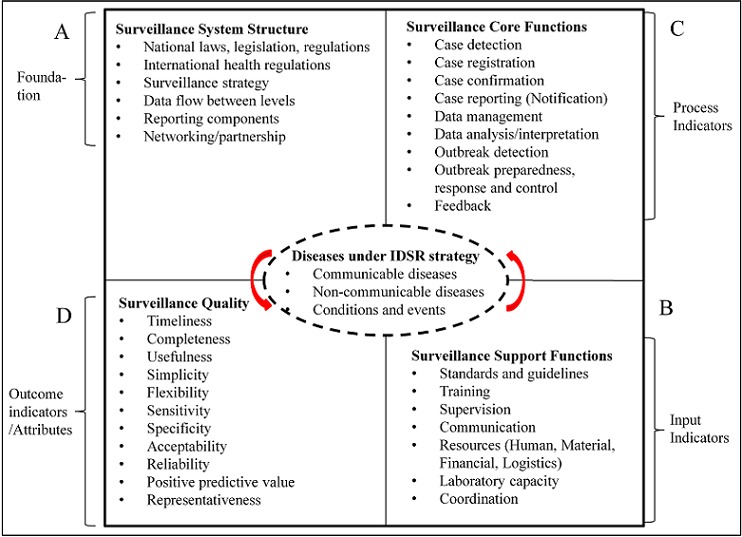
Framework for cholera surveillance under IDSR strategy study

We used semi-structured key informant interviews, field visits coupled with review of literature and government documents to frame the study into four main tenets including structure of the health system, surveillance core functions, surveillance support functions, and surveillance quality/attributes (Figure 2). The WHO-AFRO and CDC first and second editions of the IDSR Technical Guidelines [12, 14]; the WHO-AFRO regional strategy for communicable diseases 1999-2003 [11]; the IDSR standard matrix for integrated surveillance functions and skills [22]; and the WHO conceptual framework of surveillance and response systems [23] were used to design the study (Figure 2). The system structure constituted the reporting components and data flow among levels, IHR, and surveillance strategy. For the surveillance core functions, case detection, case reporting, outbreak detection, and feedback were assessed. Support functions included standard guides, training, supervision, resources, and laboratory capacity. Surveillance attributes included completeness (proportion of reporting stations that submitted complete surveillance data to the next level), and timeliness (proportion of surveillance data submitted on time) of reporting (the others were not assessed).

### Data collection

For primary data, semi-structured interviews were administered, in person, following a hierarchical strategy. Information about support functions came from the WHO country office, while key informants at the MoPH commented on surveillance system structure. Regionally, data sought at the Regional Public Health Delegations (RPHDs) and cholera command and control centers (C4) concerned both surveillance core and support functions. Data collected at the HD level centered on data analyses, outbreak investigation and reporting. At the HF level, data obtained focused on detection, reporting, laboratory process, and specimen collection. Interviews lasted about an hour-and-a-half between April 15-25, and June 22 to July 17, 2013. In total, 30 officials were involved in both regions. Secondary data come from peer-reviewed articles and government reports. We searched the PubMed, web of sciences, and google scholar databases using the key words “Cameroon”, “Cameroun”, “cholera”, “integrated”, “disease surveillance”, and “response”. Further searches were conducted on the CDC [24], WHO-AFRO [25] and MoPH [26] websites and google with these same key words.

## Results

### Surveillance system structure of cholera *National and international health laws and regulations*

**Figure 3:**
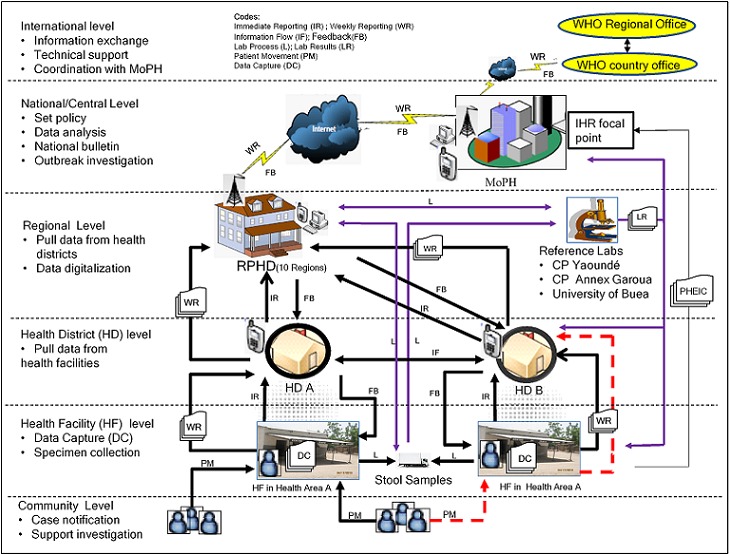
Structure of health and cholera surveillance data flow in Cameroon

In 1982, Decree No. 82-589 of 20th November 1982 created operational dialogue structures at the central, regional and periphery levels. In 1995, a decree was adopted to reorganize periphery health services into health districts. The Decree No. 87/529 of 21st April 1987 allowed private hospitals to play the role of district hospitals [27]. Article 43 of presidential Decree 2002/209 of October 2002 guides the coordination of epidemiological surveillance in Cameroon and abroad. Resolution WHA 58.3 adopted the IHR (2005), with expanded scope from yellow fever, plague, and cholera to all public health emergencies of international concern. IHR (2005) came into full force in Cameroon in 2007 [28], and since it requires a reliable surveillance system that provides data to the central level, the IDSR is suited for its implementation in Cameroon. To this effect, an IHR national focal point was designated at the MoPH (Figure 3).

#### Surveillance strategy, data flow between levels, and reporting components

The IDSR-strategy was known at the central and regional levels, but was less known at the levels below them. The reporting week runs from Friday to Thursday; HFs report weekly aggregate data to HDs every Friday. On Mondays HDs merge the data and forward them on Tuesdays in paper format to the RPHD. The data must reach the MoPH via internet latest 12 PM on Thursdays. On Fridays, analyzed data are presented to the surveillance staff, and from there are sent to the WHO. Thus, these reporting components perform specific duties. The WHO-country office in the capital Yaoundé (international level) provides support to implement IDSR, and works in collaboration with the WHO Regional Office in Brazzaville, Congo (Figure 3). MoPH (national/central level) coordinates with WHO country office. The IDSR surveillance unit is located at the Department of Disease Control and Prevention under the MoPH. RPHD (intermediate/regional level) buttresses technical support to the HD, health area (HA), HF and the community (periphery level). The periphery operationalizes public health surveillance programs (bottom of Figure 3). The HD is a geographical entity serving between 50,000 to 300,000 inhabitants with a district hospital. In 2008, there were 178 HDs, which increased by 2.7% to 183 in 2013. Each HD is broken down into HAs, which increased by 11.1% from 1,587 in 2008 to 1785 in 2010 [29]. A HA is a geographical entity organized around a HF, typically an integrated health center (IHC). Table 1 presents the regional distribution of HDs, HAs, and HFs in 2010. Under the IDSR, HF includes institutions with outpatient and/or in-patient consultation services. These include general hospitals (1^st^ category hospitals at the central level); central hospitals (2^nd^ category hospitals at the central and regional levels); regional hospitals (3^rd^ category hospitals found at the regional level); district hospitals (4^th^category hospitals at the HD level); district medical centers, and IHCs (5^th^ category hospitals found at HA level). Public IHCs decreased from 1,888 by 4.61% in 2008 to 1,801 in 2010 [29]. Epidemiological cholera data collection (see Figure 3, DC-data capture) happens at the HF when patients presents with watery diarrhea from the community (indicated in Figure 3 as PM-patient movement). The community is reprSurveillance core functions presented by basic village-level services, which refer suspected cholera patients to HFs.

**Table 1:** Regional distribution of health areas, districts, and facilities as of 2010

Region	HA	HD	Public IHC	Private HF	DMC	DH	HF (other)	RH	CH/GH	Total # of HFs
Adamawa	72	8	79	39	8	7	3	1	0	137
Centre	291	31	284	230	39	29	40	0	6	628
East	113	14	115	35	17	13	9	1	0	190
Far North	263	30	262	55	19	22	8	2	0	368
Littoral	147	19	142	115	18	18	21	2	2	318
North	159	15	127	30	5	11	15	1	0	189
North West	206	18	182	102	20	16	3	1	0	324
West	234	20	316	177	27	20	17	1	0	558
South	109	10	132	55	13	8	14	1	0	223
South West	191	18	162	60	15	12	28	2	0	279
Total	1785	183	1801	898	181	156	158	12	8	3214

Abbreviations are as ensuing: **HA** (Health Area), **HD** (Health District), **IHC** (Integrated Health Centre),**HF** (Health Facility), **DMC** (District Medical Centre), **DH** (District Hospital), **RH** (Regional Hospital),**CH**(Central Hospital), **GH** (General Hospital). Note: In 2010, there were 28 and 30 HDs in the Far North and Centre regions, respectively, but in 2013, two were added to the former and one to the latter. Source; Ministry of Public Health (MoPH). National Health Plan (NHP) 2011-2015. Republic of Cameroon. Ministry of Public Health. 2015:1-172 [Translated from French].

### Surveillance core functions

Table 2 provides an overview of the surveillance system levels with respect to the core functions. The Cameroon cholera public health surveillance system within the IDSR strategy is passive surveillance in which public health officials wait for reports from HFs [30]. However, the passive system turns into active surveillance when an outbreak is confirmed [31].

**Table 2:** Integrated Disease Surveillance and Response (IDSR) standard matrix for integrated surveillance functions and skills by health system level in Cameroon

Level	Detect	Report	Analyze and Interpret	Investigate and confirm
Commmunity	Use simple case definition for suspected cases: Y.	Report info to HFs: P	N/A	N/A
Health Facility	Use standard case definition, collect & transport specimens for lab confirmation: Y.	Report case-based info to next level: Y.	N	Investigation: P; Collect, store, &transport stool samples for *V. cholerae* lab confirmation: Y.
Health District	Collect & review data quality from HFs: Y; Ensure reliable supply of data collection & reporting tools: P; Collect & transport samples for lab confirmation: Y.	Ensure HFs use standard case definition: Y; Report data timely: P; Ensure HF staff knows when and how to report suspect cases: P.	Define denominators & ensure their quality: Y; Aggregate data from HF reports: Y; Analyze data by place, time, and person: N	Lead investigations: P; Assist HFs safely collect & transport samples: Y; Receive & interpret lab results: Y; Ensure reported outbreak is confirmed: Y; Report confirmed results to RPHD: Y.
Region	Data quality review: Y; Ensure reliable data collection & reporting tools : P; Collect & transport stool for lab confirmation: Y; Use local labs to confirm suspected cases: N.	Ensure HFs know and use standard case definition: Y; Ensure HF staff know when and how to report cases: P; Report lab results & data on time: Y	Define denominators & ensure their quality: Y; Aggregate data from HF reports: Y; Analyze data by place, time, and person: P; Calculate rates: Y; Describe risk factors: Y	Lead investigations: P; Assist HFs safely collect & transport samples: Y; Receive & interpret lab results: Y; Decide if reported outbreak is confirmed: Y; Report confirmed results: Y.
Centre	Define, update & ensure compliance with national policy & guidelines: Y; Use national lab for confirmatory and specialized testing: Y.	Report outbreaks timely to appropriate authorities: Y; Inform WHO as indicated by IHR (2005): Y.	Set policies & procedures: Y; Analyze/interpret data: Y; Meet regularly with technical coordinating committee to review analyzed & interpreted data before wide dissemination: Y.	Ensure guides for outbreak investigation in all sites: P; Share info& collaborate with int’l authorities: Y; Alert and support lab participation: Y; Provide logistics support; Y; Epidemic response team: Y.
**Surveillance functions continued**				
**Level**	**Evaluate**	**Feedback**	**Respond**	**Prepare**
Commmunity	N/A	N	Partake in response activities, behavior change education: Y	N
Health Facility	N	N	Participate to manage contact cases: Y.	Conduct training of community: Y.
Health District	Conduct regular supervisory visits: P	Alert nearby districts about outbreaks: Y; Give HFs regular and periodic feedback on routine control & prevention: Y; Data quality feedback: N	Select and implement appropriate public health response: Y; Plan timely info & education activities: P.	Support and conduct HF-base surveillance: Y.
Region	Conduct regular supervisory visits: NR.	Alert nearby districts about outbreaks: Y; Give HFs regular & periodic feedback on routine control and prevention activities: Y; Give feedback on surveillance & data quality finding:N	Implement appropriate response: Y; Plan timely education activities: P; Convene ERC & plan response: Y; Emergencytraining: Y; Plan community education: P; Alert neighbors of outbreaks: P.	Participate in EPMC: Y; Conduct training exercises for staff: Y, Conduct risk mapping & potential hazards: P; Support and conduct HF-based surveillance: Y, Organize & support rapid response team: Y.
Centre	Monitor IDSR & lab core indicators regularly: Y; Conduct outbreak investigation after action review: Y; Conduct IDSR regular review meetings: NR; Conduct regular supervisory visit: NR	Distribute bulletin for epidemiology and public health: Y; Give districts regular periodic feedback about routine control and prevention activities: Y; Release info quickly, transparent manner & listen to the affected community: Y.	Set policies, procedures for response to cases and outbreaks: Y; Support epidemic preparedness and rapid response including rapid response teams: Y; Report and disseminate results of outbreak response in bulletins, media, press releases and briefings: Y.	Set policies & training strategies: Y; Adapt & distribute risk-maps: Y; Develop messages for community education: Y; Organize & support national rapid response teams: Y. Establish & maintain public health emergency command & operations center: Y

Codes: Y = Activity being done; P = partially done activity; N/A = Not applicable; N = Many activities not being done; NR = Not regularly done activity; info = information; HF = Health Facility; HD = Health District; IHR = International Health Regulation. ERM = Emergency Response Committee. EPMC = Emergency Preparedness and Management Committee. Source; Perry HN, McDonnell SM, Alemu W, Nsubuga P, Chungong S, Otten MW, Lusamba-dikassa PS, Thacker SB. Planning an integrated disease surveillance and response system: a matrix of skills and activities. BMC medicine.2007; 5(24):1-8.

### Case detection, registration, and confirmation

Case definitions: a suspected case is anyone presenting with acute diarrhea with or without vomiting, with profuse diarrhea (10 to 100 stools/day) that looks like rice water or palm wine, with rapid dehydration or death because of acute watery diarrhea. A suspected case becomes a confirmed case with laboratory isolation of *V. cholera*. One laboratory confirmed case of cholera is the threshold to declare cholera epidemic. This definition was presented on request during interviews. However, record review showed it was changed in 2010 [32] to, A suspect is any patient aged ≥ 5 years with severe dehydration or death from acute watery diarrhea. During an epidemic, a suspected case is any person aged ≥ 5 years with acute watery diarrhea, with or without vomiting. A confirmed case is a suspected case in which *V. cholera* O1 or O139 has been isolated from the stool. At the community level, cholera is defined simply as any person age ≥ 5 years with lots of watery diarrhea. Case registration at HF is by nurses who enter case data (age, sex, symptoms, etc.) in a hospital register (paper forms). Stool samples are taken, put in a leak proof boxes, and sent directly or via the district office and/or RPHD to the laboratory for confirmation (Figure 3, L-lab process). There are no laboratory services at HFs to confirm cholera cases and outbreaks.

#### Case reporting

At the community level, community health workers (CHWs), traditional healers and birth attendants, veterinarians, pharmacists, and religious leaders report suspected cases and community deaths to HF. At HF level, two mechanisms of reporting are noted—immediate reporting and weekly reporting. As such, nurses report suspect cases to the HD office immediately (Figure 3, IR-immediate reporting) in paper format (accept the North and Far North Regions). Then aggregate cholera data is reported from HF to HD weekly by paper format (Figure 3, WR—weekly reporting). So immediate reporting is to trigger immediate case-based investigation, while weekly reporting involves the aggregate data of cases and deaths, which are used in trend analysis. Zero reporting, weekly reporting of “zero cases” when no cases are detected, is also practiced. At HD level, general administrators report data to the RPHD immediately by mobile phone ensued by weekly reporting in paper format. Immediate and weekly reporting from the RPHD to the MoPH are by phone and internet, respectively (Figure 3). In turn, epidemiologists at the MoPH report the routine weekly aggregate data to the WHO country office (Figure 3).

By default, patients are supposed to visit a HF of their HA, which the registers will indicate the patient visited HF where they live. This information is crucial for case-by-case investigation. On the left of Figure 3, patients are presenting to the HF of their HA, which falls under, say, HD A. However, some patients visit HF out of their HA (dashed red arrow, Figure 3). In this instance, patients belonging to HA A present to HF in another HA. The patients’ information are captured in the other HA and reported to HD B (dashed red arrow, Figure 3). Then HD B would immediately inform HD A by phone of receiving patients of their HA. HD A would then dispatch a team of investigators (physicians, district health officers, epidemiologists) to its HA for investigation. However, weekly aggregate data of the patients are not reported to HD A, rather they are reported directly to RPHD.

#### Data management and analysis

Surveillance data management and analysis tools including case-based reporting forms and computers were not available at HF and HD levels. Two out of the eight HDs visited lacked back-up copies of the data sent to the RPHD. There is no data analysis and interpretation at the HF (except for Kolofata district hospital, Far North) and HD levels. Data are digitalized and de-identified at the RDPHs but not analyzed. Data are analyzed at the MoPH by measures of time, place, person, and visualized using tables, graphs, and maps. Analyzed data are presented to the minister of public health on Mondays for action.

#### Outbreak detection, preparedness, response, and control

Trends and peaks are interpreted at the MoPH to detect outbreaks, assess public health impact, and plan interventions. Preparedness for immediate investigation of suspected outbreaks is weak at the HF and HD but strong at the regional level because of C4. Preparedness plans, supplies (2% chlorine, cholera vaccines, oral rehydration salts), and equipment (rapid test kits) were not maintained for immediate action at the latter levels. Without vaccines stocks, the option of vaccination is not present at any level. There is no rapid outbreak response team at HF and HD levels. Materials for community education (handwashing, safe drinking water and storage, food preparation and handling, safe burial practices, safe disposal of waste, and infected clothing) and treatment plans were available on walls at HF, HD, and RPHD levels. These were presented on request. Control activities including disinfection of wells, and restriction of mass gatherings (funerals and weddings) besides alerting neighboring locations are performed by C4, central, and WHO country office (depending on the scale of outbreak).

#### Feedback and dissemination

There is no feedback bulletin from RPHD to HD and from HD to HF. However, written and verbal reports are given at staff meetings. Furthermore, there is a national bulletin for dissemination namely the Epidemiological Bulletin of Cameroon commonly known by its French acronym BEC (Bulletin épidémiologique du Cameroun) [17]. BEC disseminates data on completeness and timeliness of reporting to RPHDs and HDs but not to HFs. Outbreak response reports are also provided.

### Surveillance support functions *Standards and guides, training and supervision, and laboratory capacity*

The first and second editions of Technical Guidelines for the IDSR strategy were presented upon request at RPHD, but not at HD and HF levels. The technical guide for the “zero charge” telephone fleet was found at C4 of the Far North only. Training in surveillance is weak at the community, HF, and HD levels; but strong at the regional, and central levels. Supervisory visits are only partially executed by RPHD and HD levels. The HF, HA, and community levels do not perform supervision.

**Figure 4:**
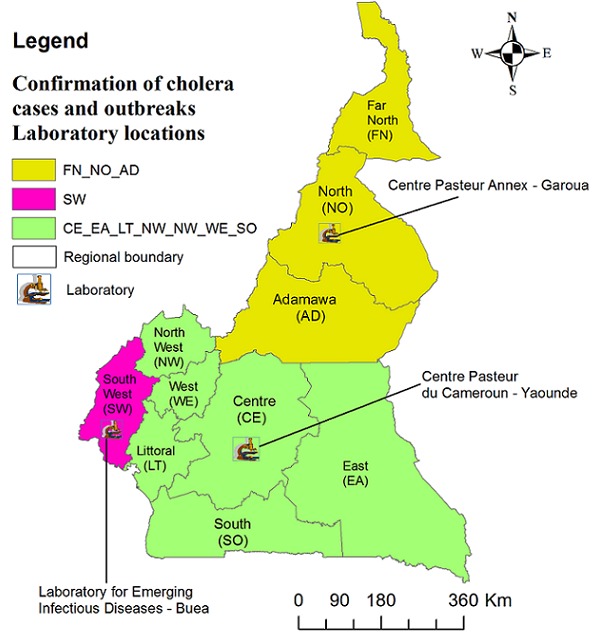
Laboratory locations for the confirmation of *V. cholerae*

HFs and HDs (except the Kolofata district hospital) do not have lab capacity to confirm cholera case and outbreak. However, three reference laboratories situated in three regions serve to confirm *V. cholera* cases and outbreaks (Figure 4). These labs are well equipped for microbiological testing of *V. cholerae* and sero typing. They also have standby generators, a good cold chain, and enough storage capacity for long-term conservation of *V. cholera* strains.

#### Resources (human, material, and financial) and coordination

Epidemiologist, public health workers, physicians, nurses, and CHWs perform surveillance activities at various levels. There was evidence of a vehicle at the regional level for field investigation. Financial support for the adoption of IDSR strategy comes from the MoPH, WHO, Global Viral Forecasting Initiative, and other partners.

**Figure 5:**
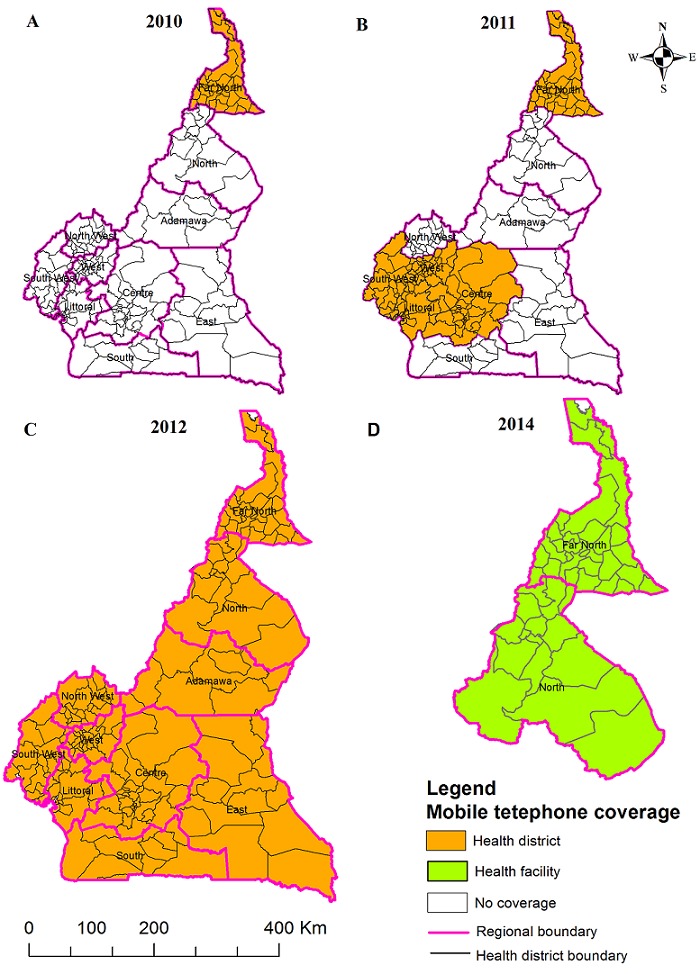
Evolution of mobile telephone coverage for cholera surveillance

In 2010, mobile phones were introduced for the first time at the district level in the Far North only. Figure 5 A-D shows the evolution of the mobile phone coverage in the country. First funders of this initiative were the United States embassy in Yaoundé followed by the European Union, and thanks to the African Development Bank, disease phone reporting at “zero charge” became a reality. IDSR refers to the latter as “green line” telephone. As an incentive to always carry the phones, calls for private issues out of the network are made at half the standard charge. The incentive is designed to motivate the operators to use the phones for both their private and on-duty needs. Otherwise, users would have to carry two different phones, which could demotivate disease reporting. Their functions are not limited to immediate reporting of suspected cases of cholera. An example of how the mobile phone surveillance system helped to prevent and control the spread of cholera through funeral rituals during the 2010-2012 outbreaks was explained during field visits. This involved a dead body that was being transported from one region (A) to another region (B) for burial. Region A sealed the casket with instruction not to be opened (at any of the stops on its way to burial in region B) to perform the funeral ritual of touching corpses. To ensure compliance, WHO country office used the mobile phones to notify the RPHD and the health district officer (HDO) in B about immediate burial of the corpse on arrival without opening the casket. HDO of B contacted the family concerned and the diseased was buried without opening the casket (WHO country office). Thus, preventing the spread of cholera by hindering the traditional funeral ritual of touching corpses was possible thanks to the availability of the mobile phones at the district level.

For co-ordination, the MoPH and WHO country office created the operations Cholera Command and Control Center (C4) between 2010 and 2011 in the Far North region to provide immediate alert to new outbreaks. C4’s role is to offer technical coordination to partners in the context of epidemiological and laboratory surveillance, mobilization, case management, logistical support, and infection control [33]. Today, C4 operates in all 10 Regions of Cameroon.

### Surveillance quality/attributes *completeness and timeliness of reporting*

In 2010, completeness was from the regional to the central level only—95% (range 38.46-100%), and timeliness was 0% [34]. In 2011, completeness was 97% (range 79-100%) at the central level and 83% (range 48-99%) at the regional level, and timeliness was 33% (range 4-81%) at the central level, and 23% (range 0-50%) at the regional level [35]. In 2012, completeness at the central and regional levels was >90% whereas timeliness rose to 78% (range 54-96%) and 60% (range 41-85.8), respectively [36].

## Discussion

### Strengths

Cholera under the WHO-AFRO-IDSR strategy is an epidemic-prone threshold disease with alert (one suspect case triggers prompt case-based reporting and specimen collection) and epidemic (one lab confirmed case triggers epidemic declaration) thresholds. These were constantly stressed by our key informants at the MoPH and RPHDs and so show mastery of the IDSR strategy of early detection and rapid response; and thus, strength in its own merit. Important improvements in the hierarchical structure towards integration and achieving early detection and rapid response in cholera surveillance are in effective use. This is important because various levels in the hierarchy will have the information they need for evidence-based decision-making. The availability of “green line” mobile fleet in all HDs and recently in HFs in the North and Far North Regions [37] is one of the greatest milestones in the IDSR strategy in Cameroon. The application of the “green line” mobile surveillance system in improving immediate reporting and co-ordination of prevention operations was evident during field visits. Besides the provision of laptops at the regional level; the creation of 183 health districts; and C4 in all 10 Regions; and the availability of vehicles (in some RPHDs) for investigation are important milestones in facilitating surveillance core and response functions. These are laudable efforts and improvements in cholera public health surveillance, especially as studies elsewhere also show important improvements towards IDSR strategy implementation [38]. Despite these encouraging achievements, gaps remain which if diligently exploited could help the system operate at its maximum potential.

### Challenges of the surveillance system

The goal of the IDSR strategy of data analysis and rapid response at the district level is hindered by many challenges. First, the lack of “green line” mobile phones at HFs (except the North and Far North Regions) might delay immediate reporting of suspected cases, which might be inadequate to arrest the explosive takeoff of epidemics. Extending the “green line” fleet to all HFs is necessary. Secondly, HFs (except Kolofata district hospital [32]) and HDs do not perform data analysis and interpretation. RPHDs do not perform data analysis and interpretation either despite the presence of computers. Hence, specific health area and district level cholera trends might not be timely available for decision making in light of early detection and rapid response at the HD level as stipulated by the IDSR strategy. The lack of data analysis at the district level is not just an issue in Cameroon but also in Tanzania [39] and other countries in Africa implementing the IDSR strategy [40]. Computers and trained surveillance personnel at the district level will be a great boost to the IDSR strategy. Thirdly, nurses at HFs and general administrators at HDs perform data processing by sending paper forms to the RPHD where epidemiologist or other staff digitalizes the data. Staffing the HDs with data entry operators and shifting the data digitalization to the HD level would be consistent with IDSR strategy. An ideal situation would be to shift data digitalization to HFs, which would improve data quality, reduce the burden, and cost of paper reporting; but this might not be feasible in a resource scarce setting. Besides studies show that when health care providers take over surveillance activities, their workload increases, resulting in fatigue and demotivation [41]. Perhaps this work overload, also underscored by Djomassi *et al.* [30] and Nsubuga *et al* [13], results in low timeliness and completeness of reporting of routine data from the HD to the RPHD. Fourthly, cholera case definition was changed in 2010, but the outdated case definition was handed to us during our field visits. It is very likely that the system is not putting to full use the updated case definition. More supervision at various hierarchical levels is needed to ensure the use of up-to-date materials. Fifthly, the IDSR strategy recommends each hierarchical level to have a functioning laboratory. Regrettably, HFs lack labs and/or existing ones are ill equipped. Equipping HF labs would be in tune with early detection, confirmation and rapid response. Perhaps the ISDR strategy was overly ambitious in recommending labs at all levels in a resource rare setting.

All the above-mentioned deficiencies show that despite major improvements in IDSR strategy implementation, much still needs to be done for better cholera surveillance in Cameroon.

### Limitations and future direction of study

This study presents cholera surveillance within the IDSR strategy from the perspectives of key informants in two out of ten regions of Cameroon. The choice of these two regions was determined initially by the frequency of cholera epidemics there and secondly by the availability of focal persons that were vital in providing essential information. As such, selection bias can not be ruled out. The Centre region was particularly chosen because it is the capital and therefore, contains the MoPH that oversees into the national health policy. Other regions of this country could have trends that vary from the findings in these two regions. Future studies could compare the IDSR strategy core activities and support functions in the northern Regions with Muslim culture and the southern regions with Christian culture. Studies comparing the costs of paper based reporting from HFs to the RPHDs and electronic data digitalization at HFs could provide the needed direction in decision-making. The IDSR strategy seeks to merge single disease surveillance systems into an integrated multi-disease surveillance system with response at the district level [19]. Cholera is just one among them and we have no understanding of the other diseases under this strategy. Expansion into the IDSR surveillance of the other diseases will improve our understanding of how synergy is leverage to achieve communicable disease integrated public health surveillance goals of early detection and timely intervention to reduce morbidity, disability, and mortality.

## Conclusion

Cholera outbreaks are a threat to public health in Cameroon. Cameroon’s adoption of the WHO-AFRO-IDSR strategy in 2003 was critical for early detection and rapid response to prevent cholera. However, the lack of “green line” mobile phone surveillance system (except North and Far North) might not be in tune with early detection and rapid response to outbreaks. Equipping all HFs with the “green line” mobile surveillance approach is here called for. While surveillance core, response, and support functions were well articulated at the MoPH and RPHD, they were, however, less known at the levels below them. In addition, cholera case definition changed in 2010 but limitedly distributed. Education and more supervision are needed to ensure the use of updated information and materials. HFs and HDs lack surveillance personnel, computers, and labs are ill equipped. These could hamper immediate reporting of cases and delay the confirmation of outbreaks. Equipping the HFs and HDs with these materials will improve reporting and confirmation of outbreaks. There is almost no data analysis and interpretation at HF, HD and RPHD. Feedback, training and supervision are strong at the MoPH but week at the latter levels. Completeness and timeliness varies considerably among levels. Thus, the goal of WHO-AFRO-IDSR strategy of data analysis and rapid response at the district level has not been met. Both human and material resources are needed at the HD level to achieve this goal. We hope that the findings from this in-depth assessment of cholera public health surveillance would help to guide improvements in the surveillance system in Cameroon and beyond.

### What is known about this topic

- Cameroon first adopted the Integrated Disease Surveillance and Response (IDSR) strategy in 2003, which ended the single disease program that relied on vertical public health surveillance systems;
- First and second editions of the Technical Guidelines were implemented in 2005, and 2011, respectively;
- Cholera is one of 47 diseases under IDSR surveillance in Cameroon; Cholera surveillance system is passive, but turns active during outbreaks.

### What this study adds

- Detailed description of the flow of cholera surveillance data supported by the creation of cholera command and control centers in all ten Regions, the presence of computers at Regional level, and use of mobile phones (“greenline” mobile surveillance) at Health Districts. Case definition changed in 2010, yet the outdated version was in use in 2013;
- The cholera surveillance system lacks “green line” mobile phones, computers, surveillance trained personnel and laboratory capability at health facilities and there is no data analysis at Health Facility, Health District, and Regional levels with minimal feedback and supervision. Timeliness and completeness of reporting of routine data are low from the Health Facility to Health District and to the Regional level;
- The goal of IDSR strategy of early detection, data analysis, and rapid response at the Health District level is a challenge. Trained surveillance personnel and computers are needed at the Health District level to achieve this goal.
